# The predicting factors for indication of surgery in patients with hemoperitoneum caused by corpus luteum cyst rupture

**DOI:** 10.1038/s41598-021-97214-6

**Published:** 2021-09-17

**Authors:** Mi Ju Kim, Hyun Mi Kim, Won Joon Seong

**Affiliations:** Department of Obstetrics and Gynecology, Kyungpook National University Hospital, School of Medicine, Kyungpook National University, 807 Hogukro, Buk-gu, Daegu, 41404 Republic of Korea

**Keywords:** Risk factors, Signs and symptoms

## Abstract

The aim of the study was to determine the risk factors for surgery in patients with hemoperitoneum caused by corpus luteum cyst rupture. A retrospective review of medical records of 155 patients diagnosed with hemoperitoneum caused by corpus luteum cyst rupture was conducted between January 2010 and March 2015. The patients were divided into two groups: surgical and conservative management. The differences in characteristics between the two groups were compared. The indicators that determine the need of a surgery at the initial visit were also compared between the two groups. Initial hemoglobin level was lower (11.3 ± 1.4 g/dL vs. 12.2 ± 1.2 g/dL; *p* = 0.007) in the surgery group. There were significant differences in posterior cul-de-sac (PCDS) fluid collection depth (6.2 ± 2.5 cm vs. 4.5 ± 1.6 cm, *p* = 0.000), total fluid collection depth (8.4 ± 1.8 cm vs. 6.5 ± 2.1 cm, *p* = 0.000), single deepest pocket depth (6.7 ± 2.2 cm vs. 5.1 ± 1.5 cm, *p* = 0.006), liver-dome fluid (78.9% vs. 35.6%; *p* = 0.002), and estimated intrapelvic bleeding amount (325 ± 250 cc vs. 206 ± 146.5 cc, *p* = 0.002). The extravasation over grade 2 was more often in surgery group (68.4% vs. 30.1%; *p* = 0.001). PCDS fluid collection depth, the presence of liver-dome fluid, and the severity of contrast extravasation through ultrasonography and computed tomography are good indicators for determining the management of hemoperitoneum resulting from corpus luteum cyst rupture in healthy women.

## Introduction

Hemoperitoneum is accumulation of blood in the pelvic cavity, which causes peritoneal irritation symptoms, such as nausea, vomiting, abdominal pain, and dizziness^1^. There are many gynecological causes of hemoperitoneum, including rupture of ectopic pregnancy or ovarian tumors, such as endometrioma. Recently, it has been reported that deep infiltrating endometriosis may also weaken the pelvic vessel related to chronic inflammation, causing spontaneous life-threatening hemoperitoneum^2–4^. The rupture of a functional ovarian cyst, such as a corpus luteum cyst, is common not only in patients with coagulopathy^5^ but also in healthy individuals with no underlying diseases.

There are two treatment options for hemoperitoneum caused by corpus luteum cyst rupture (CLCR): immediate operation or conservative treatment. More than 80% of hemoperitoneum resulting from ovary cyst rupture required surgical intervention through laparoscopy or laparotomy in the past^6,7^. However, there are demands for less invasive treatment to manage hemoperitoneum with close observation of the amount of intraabdominal bleeding, monitoring of vital signs, such as blood pressure and heart rate, and workup of the progression of anemia. If the amount of active bleeding in the pelvic cavity does not increase, the corpus luteum cyst will decrease in size, and intraabdominal blood collection will be gradually absorbed. The surgical approach is intended to remove the ovarian cyst and blood clot in the pelvic cavity. It also results in unintentional damage of normal ovarian tissue through thermal injury, which can lead to decreased ovarian reserve. In addition, surgery can result in intrapelvic adhesions around the ovaries or uterus. The skin incision site for laparotomy or laparoscopy brings with it a cosmetic consideration. While making a decision whether to operate or not, several factors should be considered on a case-by-case basis.

The risk factors of surgery for hemoperitoneum caused by CLCR were determined through a patient’s medical history, vital signs, physical examinations, ultrasonographic findings, computed tomography (CT) images, and blood sampling results at the time of the initial visit to the hospital.

## Methods

### Study design

A retrospective review of medical records of 155 patients diagnosed with hemoperitoneum with a gynecological disease at Kyungpook National University Hospital in Daegu, South Korea was conducted between January 2010 and March 2015. Most patients were presented with complaints of acute lower abdominal pain; nausea, vomiting, dizziness, or loss of consciousness as the primary symptoms seen among patients. In the emergency room, the patients’ vital signs were checked, and they underwent laboratory tests, ultrasonography, and abdomen–pelvis CT. This study included 92 patients who were diagnosed with hemoperitoneum with a CLCR on ultrasonography and/or CT. Diagnosis was reconfirmed by an expert gynecologist or radiologist retrospectively to achieve more accurate results. Sixty-one patients with hemoperitoneum resulting from ectopic pregnancy rupture; arteriovenous malformation; ovary cancer; or uterine anomaly through urine human chorionic gonadotropin test, ultrasonography, or CT images were excluded from the study. Two patients with abnormalities of coagulation were also excluded. The study participants were divided into two groups: surgical and conservative management. The differences in characteristics between the groups as well as the indicators of surgery at the initial visit were compared. This study was approved by Institutional Review Board of Kyungpook National University Hospital.

### Methods

**Figure 1:**
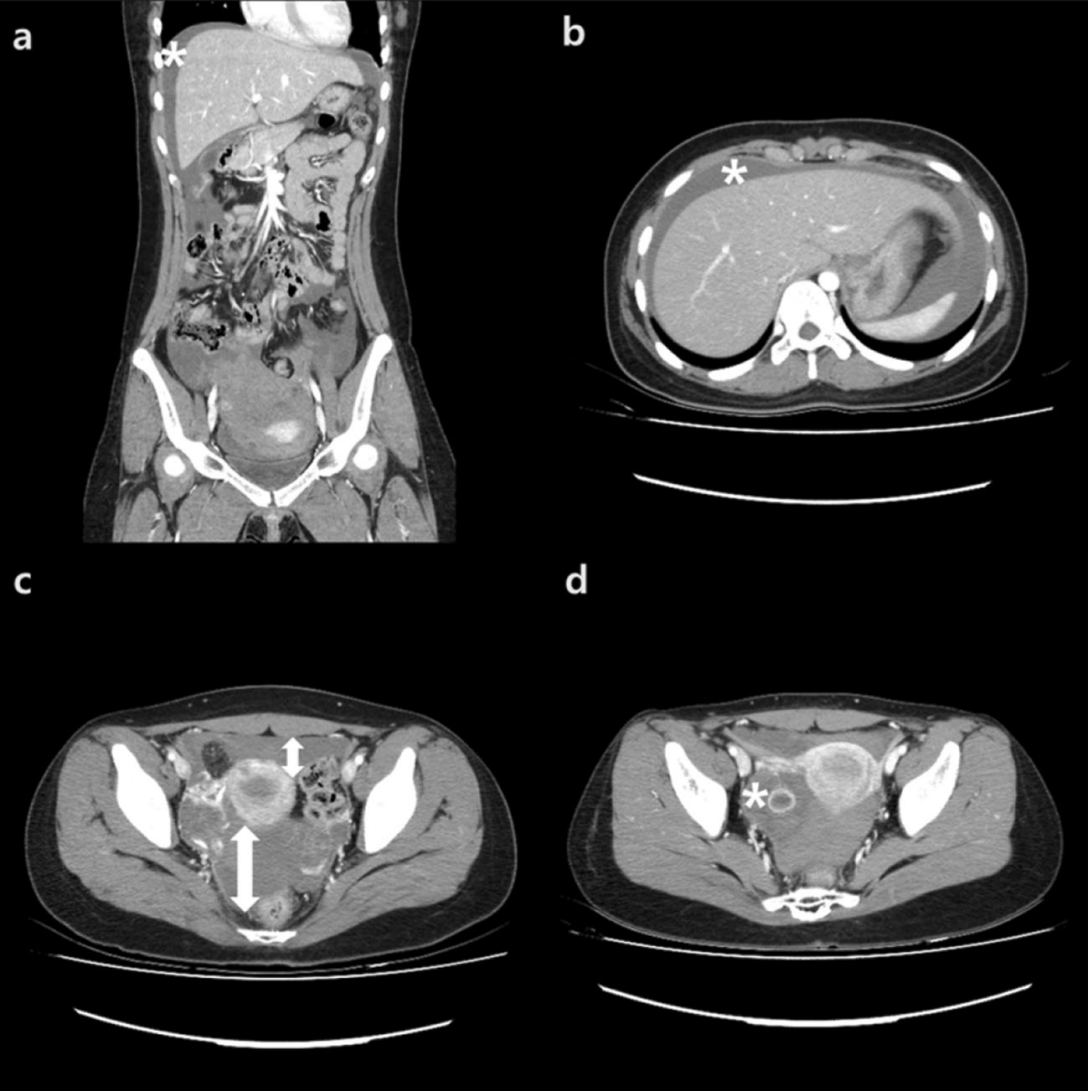
Abdomen–pelvic CT. (**a**) In coronal view, there is a liver-dome fluid collection (*). (**b**) In transverse view, fluid collection around liver-dome and stomach (*). (**c**) In transverse view, methods of anterior cul-de-sac fluid collection depth measurement, posterior cul-de-sac fluid collection depth measurement. (**d**) Extravasation from right ovary cyst rupture, hyperechogenic ring formation (*).

Patients’ vital signs, such as blood pressure, pulse rate, body temperature, and oxygen saturation were checked at the initial visit. Patients’ medical and surgical history, including menstruation, were recorded, and a physical examination was conducted by a gynecologist. If the urine human chorionic gonadotropin test was negative, ultrasonography and enhanced abdomen–pelvis CT were performed within 1 h for all patients with suspected hemoperitoneum resulting from CLCR. Transvaginal or transrectal ultrasonography was performed to know the size and location of the cyst, the estimated amount of fluid in the pelvic cavity using a generic volume program (Voluson 730; GE, Austria, Tiefenbach). Transvaginal ultrasonography was performed in patients with sexual experiences, and transrectal ultrasonography was performed in patients without sexual experiences. The measured values were repeated at least three times to determine the average value. An abdominal probe was used to locate the blood collected around the liver-dome and posterior side of the kidney, which indicated more than moderate amount of bleeding in the pelvic cavity. Enhanced abdomen–pelvic CT was performed to check active vascular leakage and to locate other possible surgical abdomens. CT images were retrospectively evaluated by a radiologist to reaffirm the diagnostic accuracy. Anterior cul-de-sac (ACDS) depth, ACDS fluid collection vertical depth, posterior cul-de-sac (PCDS) depth, PCDS collection vertical depth, and total depth were determined to measure the amount of bleeding in the transverse view. The sum of ACDS and PCDS was confirmed through CT images (Fig. 1). Ovary cyst size and morphology were also confirmed. Contrast leakage from the ovary was read by an expert gynecologist to verify active bleeding at the time of evaluation, and the severity of leakage was confirmed as grade 1, 2, and 3 by a radiologist retrospectively. Physical examination, ultrasonography, CT scan, and blood sampling were performed in all the patients within an hour of initial visit, and surgery was considered if the initial vital sign was unstable. Intravenous hydration and absolute bed rest were advised if the vital signs were stable, and the continuous monitoring of vital sign was done. Transabdominal and transvaginal/transrectal ultrasonography were repeated 6 h, 12 h, and 24 h after the first visit to the emergency room to verify the optimized amount of intraabdominal blood collection to see if CLCR bleeding continues. After 12 h and 24 h of the first sampling, hemoglobin level was checked to confirm if anemia was in progress. During the conservative treatment, surgery was performed considering a number of factors, such as deterioration of vital signs, increased size of hemoperitoneum on ultrasonography, deteriorating anemia, or worsening abdominal pain. Corpus luteum cysts were confirmed pathologically in patients of the surgical group. The differences of days requiring parenteral analgesia, hospital period, transfusion, and anemia after surgery or conservative management were compared in both groups.

### Statistical analysis

All data were analyzed using SPSS software (version 20.0; SPSS Inc., Chicago, IL, USA). Mann–Whitney *U* test was used to compare the surgically treated and conservatively managed groups for continuous numeric data, and *χ*^2^ test was used for binary categorical data. Variables with a *p-*value less than 0.05 based on the Mann–Whitney *U* test or χ^2^ test were considered statistically significant. Logistic regression analysis was performed to find independent variable to predict the necessity of surgery. Receiver operating characteristic (ROC) curve analysis for consecutive variables was performed to evaluate the cut-off value by R version 4.0.0.

### Ethical approval

All procedures performed in studies involving human participants were in accordance with the ethical standards of the institutional and/or national research committee and with the 1964 Helsinki declaration and its later amendments or comparable ethical standards.

### Informed consent

Informed consent was obtained from all individual participants included in the study.

## Results

This study included 92 women diagnosed with hemoperitoneum caused by CLCR. Of these, 19 (20.7%) were included in the surgery group, and 73 (79.3%) were in the conservative group. All surgeries in the surgical group were performed by laparoscopy. Table 1 shows the patients’ clinical characteristics. No significant differences were found between the two groups in terms of age, body mass index, marital status, parity, menstrual cycle, or pain-inducing events. Whole abdominal pain and nausea was more common in in surgery group (26.3% vs. 5.5%, *p* = 0.006; 63.2% vs. 35.6%, *p* = 0.044, respectively), but no statistically significant difference was found in rebound tenderness and the presence of loss of consciousness between both the groups. Initial vital signs showed no differences, but pulse rate was noted higher in the surgery group (97.7 ± 15.5 vs. 89.5 ± 16.0; *p* = 0.027).

**Table 1:** Clinical characteristics of patients in both groups.

	Surgery (n = 19)	Conservative (n = 73)	*p* value
Age, years	32.85 ± 4.31	32. ± 7.3	0.877
Body mass index, kg/m^2^	19.7 ± 1.8	20.8 ± 2.9	0.318
Married, n (%)	4 (21.1%)	17 (23.3%)	0.944
Nulliparity, n (%)	17 (89.5%)	58 (78.5%)	0.502
Menstrual cycle day	27.7 ± 14.7	29.1 ± 13.4	0.683
Smoking, n (%)	3 (15.8%)	7 (9.6%)	0.719
Operative history	3 (15.8%)	13 (17.8%)	1.000
Alcohol, n (%)	3 (15.8%)	14 (19.2%)	0.994
Spotting, n (%)	1 (5.3%)	3 (4.1%)	1.000
Antecedent to pain			
Any event, n (%)	13 (68.4%)	41 (56.2%)	0.445
Intercourse, n (%)	11 (57.9%)	30 (41.1%)	0.025
Whole abdominal pain, n (%)	5 (26.3%)	4 (5.5%)	0.006*
Lower abdominal pain, n (%)	14 (73.7%)	69 (94.5%)	0.160
Rebound tenderness, n (%)	16 (84.2%)	52(71.2%)	0.319
Nausea, n (%)	12 (63.2%)	26 (35.6%)	0.044*
Loss of consciousness, n (%)	1 (5.3%)	3 (4.1%)	0.845
Initial vital sign			
SBP, mmHg	113 ± 18.6	119.8 ± 2,0.5	0.134
DBP, mmHg	66.2 ± 11.1	71.9 ± 14.6	0.114
PR, per minute	97.7 ± 15.5	89.5 ± 16.0	0.027*
BT, ℃	36.6 ± 0.3	36.6 ± 0.3	0.996

SBP, systolic blood pressure; DBP, diastolic blood pressure; PR, pulse rate; BT, body temperature.

*p values < 0.05 are shown in bold with asterisk (*).

Table 2 presents the laboratory, ultrasonographic, and CT findings. The initial hemoglobin was lower (11.3 ± 1.4 vs. 12.2 ± 1.2; *p* = 0.007) in the surgery group in the laboratory testing. The other parameters did not show statistically significant differences. Regarding CT and ultrasonographic images, there were significant differences in PCDS fluid collection depth (6.2 ± 2.5 vs. 4.5 ± 1.6; *p* = 0.000), total fluid collection depth (8.4 ± 1.8 vs. 6.5 ± 2.1; *p* = 0.000), single deepest pocket depth (6.7 ± 2.2 vs. 5.1 ± 1.5; *p* = 0.006), the presence of liver-dome fluid collection (15 [78.9%] vs. 26 [35.6%]; *p* = 0.002), and estimated intrapelvic bleeding amount (325 ± 250.0 vs. 206 ± 146.5; *p* = 0.002). Although the presence of contrast extravasation from the ovaries was not significantly different (18 [94.7%] vs. 55 [75.3%]; *p* = 0.123) in both groups, the extravasation over grade 2 was more often in surgery group (13 [68.4%] vs. 22 [30.1%]; *p* = 0.001).

**Table 2:** Laboratory testing, CT, and ultrasonographic findings in both groups.

	Surgery (n = 19)	Conservative (n = 73)	*p* value
**Laboratory testing**			
Hemoglobin, g/dL	11.3 ± 1.4	12.2 ± 1.2	0.007*
WBC, /µL	10,642 ± 2051	10,740 ± 1920	0.437
Platelet, 10^3^/µL	260.7 ± 54.7	240.9 ± 50.6	0.243
INR	1.1 ± 0.9	1.1 ± 0.1	0.097
CRP, mg/dL	0.25 ± 0.11	0.29 ± 0.1	0.946
**Ultrasonographic findings**			
Laterality of ovarian cyst			1.000
Right, n (%)	12 (63.2%)	48 (65.8%)	
Left, n (%)	7 (36.8%)	25 (34.2%)	
Estimated intrapelvic bleeding amount, cc	325 ± 250	206 ± 146.5	0.002*
**CT findings**			
Size of cyst (maximal diameter), cm	3.6 ± 1	3.8 ± 1.2	0.493
PCDS fluid collection, cm	6.2 ± 2.5	4.5 ± 1.6	< 0.001*
ACDS fluid collection, cm	2.5 ± 1.1	1.8 ± 1.3	0.122
Total fluid collection, cm	8.4 ± 1.8	6.5 ± 2.1	< 0.001*
Single deepest pocket, cm	6.7 ± 2.2	5.1 ± 1.5	0.006*
Liver-dome fluid collection, n (%)	15 (78.9%)	26 (35.6%)	0.002*
Contrast extravasation from ovary, n (%)	18 (94.7%)	55 (75.3%)	0.123
Contrast extravasation over grade 2 from ovary, n (%)	13 (68.4%)	22 (30.1%)	0.001*

ACDS, anterior cul-de-sac; CRP, C-reactive protein; INR, international normalized ratio; PCDS, posterior cul-de-sac; WBC, white blood cell.

*p-values < 0.05 are shown in bold with asterisk (*).

In multiple regression analysis the odds ratio of the severity of extravasation of contrast from ruptured ovary on CT and liver-dome fluid collection was 3.487 and 4.948, respectively (Table 3).

**Table 3:** Logistic regression model.

	Odds ratio	95% CI		Significance
		Lower limit	Upper limit	
Presence of extravasation	1.111	0.178	6.952	0.910
Posterior cul-de-sac fluid	1.245	0.989	1.566	0.062
Extravasation over grade 2	3.487	1.027	11.832	0.045*
Liver-dome fluid collection	4.948	1.443	16.965	0.011*

CI, confidence interval.

*p-values < 0.05 are shown in bold with asterisk (*).

**Figure 2:**
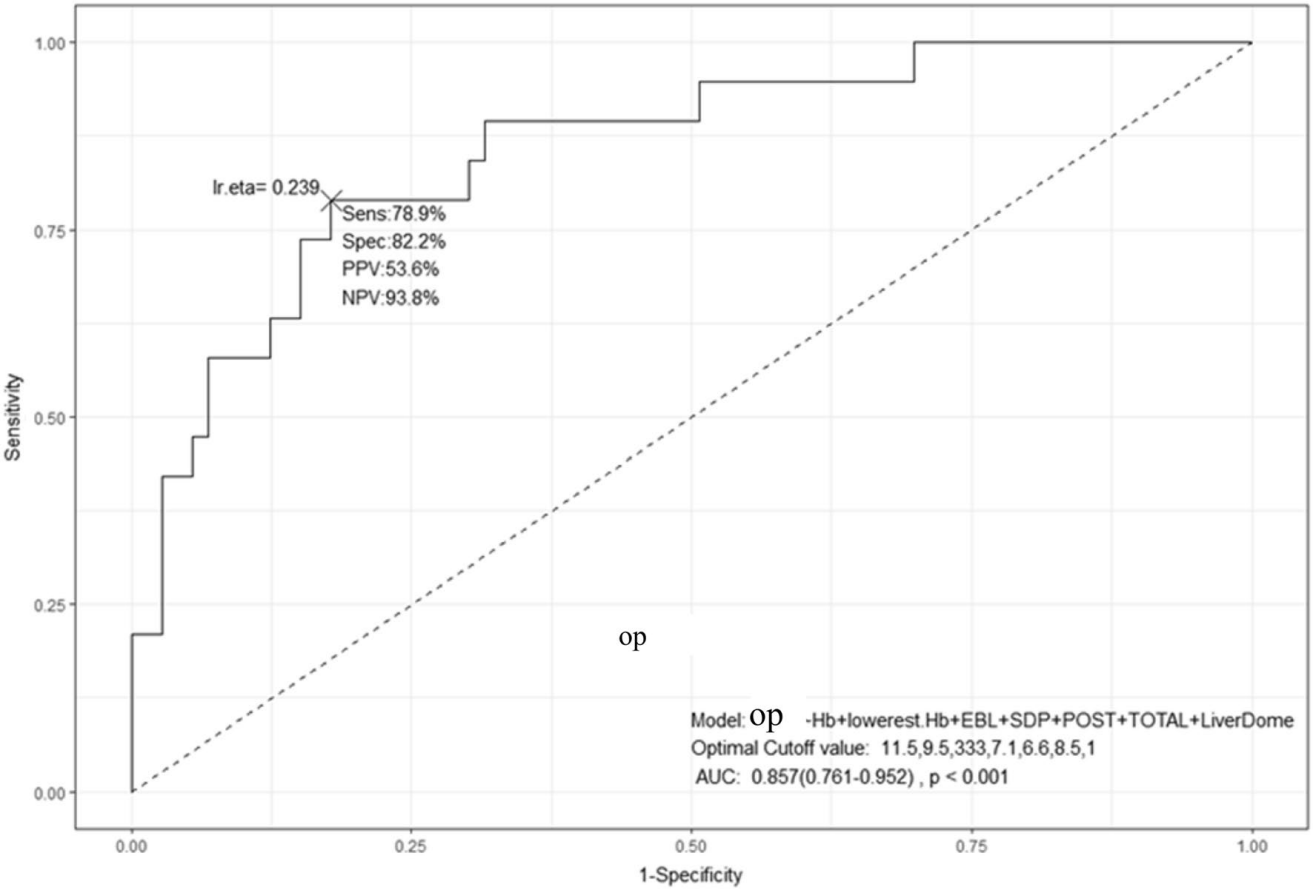
Receiver operating characteristic curve (ROC) indicating the risk factors for surgery. The ROC curve, which combines the aforementioned statistically significant factors, in initial hemoglobin, the lowest hemoglobin, estimated bleeding amount, single deepest pocket, posterior cul-de-sac fluid collection depth, total depth, and presence of liver-dome fluid, had a higher rate of prediction for surgical treatment (AUC: 0.857 [0.761–0.952, *p* < 0.001]).

On the ROC curve analysis indicating the risk factors predicting surgery, if the estimated pelvic bleeding amount is more than 225 mL, area under the curve (AUC) is 0.731 (0.609–0.853, *p* < 0.001), and if the cut-off value of PCDS fluid collection depth is more than 5.7 cm, AUC is 0.781 (0.67–0.892, *p* < 0.001). If the total fluid collection depth is more than 7.7 cm, AUC is 0.763 (0.652–0.874, *p* < 0.001), the presence of liver-dome fluid collection predicted AUC of 0.717 (0.607–0.826, *p* < 0.001). However, the ROC curve, which combines the aforementioned statistically significant factors, initial hemoglobin, the lowest hemoglobin, estimated bleeding amount, single deepest pocket, PCDS depth, total depth, and presence of liver-dome fluid, had a higher rate of prediction for surgical treatment (AUC: 0.857 [0.761–0.952, *p* < 0.001]) (Fig. 2).

Table 4 represents the hospital stay progression in both groups. There was a higher chance of anemia requiring injection of iron (13 vs. 24; *p* = 0.011) or transfusion (10 vs. 7; *p* = 0.000) in surgery group, but the days requiring parenteral analgesia and the hospitalization period were not different between the surgery and conservative groups.

**Table 4:** Hospital stay progression in both groups.

	Surgery (n = 19)	Conservative (n = 73)	*p* value
Lowest hemoglobin, g/dL	8.5 ± 1.1	10.3 ± 1.4	< 0.001*
Days requiring parenteral analgesia, days	2.1 ± 1.7	1.6 ± 0.8	0.257
Hospital stay, days	3.9 ± 1.3	3.6 ± 1.5	0.314
Hemoglobin level at discharge, g/dL	9.4 ± 0.7	10.8 ± 1.3	< 0.001*
Transfusion, n (%)	10 (52.6%)	7 (9.6%)	< 0.001*
Iron intravenous injection, n (%)	13 (68.4%)	24 (32.9%)	0.011*
Fever, n (%)	3 (15.8%)	8 (10.9%)	0.856

*p-values < 0.05 are shown in bold with asterisk (*).

## Discussion

In the past, surgical approach was used to confirm definite diagnosis, to remove the hematoma and to coagulate the focus of bleeding in patients with CLCR. Raziel et al. reported the rate of the surgical approach for CLCR was as high as 83% in 1993^6^. Imaging modalities were not as developed as it is now. Therefore, it was difficult to distinguish between CLCR and other acute abdominal diseases, such as ectopic pregnancy and appendicitis^8^. Development of better diagnostic imaging techniques increased the accuracy of diagnosis in patients with gynecological hemoperitoneum. If the vital signs are normal, and there is no more bleeding from the ovary observed, surgery is not always necessary. The need for conservative management has greatly increased as most bleeding is absorbed spontaneously within a few days without any serious complications^9^. However, if the active bleeding continues, and vital signs are unstable, surgical management is mandatory. Therefore, it is important to predict in advance which patients will need surgical treatment. Bottomley et al. suggested that the indications of surgery for hemoperitoneum include uncertain diagnosis, no relief of symptoms, increasing hemoperitoneum on imaging tests, or aggravating anemia^10^. If the need for surgery can be determined early using ultrasonography or CT, unnecessary surgical complications and problems arising from delaying surgery can be avoided.

High pulse rate and low hemoglobin at the initial visit indicated the need for surgery in this study. No significant differences were found in the size and laterality of the ovary cyst on evaluation of ultrasonographic findings. However, the estimated intrapelvic bleeding amount using a three-dimensional ultrasound was a significant risk factor for surgical treatment. PCDS fluid collection depth, total fluid collection depth, single deepest pocket depth, and severity of contrast extravasation from the ovary were indicators of surgical management in abdomen–pelvic CT, transverse and coronal views. A large amount of bleeding increases the possibility of operation, indicating severe anemia and huge hematoma in pelvic cavity, as well as an active extravasation over moderate grade (Odds ratio [OR]: 3.015 (95% confidence interval [CI]: 1.051–10.685]). The presence of liver-dome fluid also increased the need for surgery by 4.948 times.

Previous reports published the cases of hemoperitoneum caused by CLCR with various coagulopathies. Coagulation disorders included aplastic anemia^11,12^, factor V deficiency^13^, factor XIII deficiency^14^, congenital hypofibrinogenemia^15^, and immune thrombocytopenic purpura (ITP)^16^. There was a study in the conservative group using tranexamic acid as well as fluids and blood transfusion^7^. This study targeted healthy patients without coagulopathy and treated only with fluid and antibiotics for conservative treatment.

Several authors have analyzed the predicting factors of surgical management in hemoperitoneum. Kim et al. reported that a low diastolic blood pressure and a large amount of hemoperitoneum indicated the need for surgery through CT^17^. Lee et al. reported that the presence of active bleeding and hemoperitoneum depth on a pretreatment CT scan can be predictive warning signs of surgery^18^. This study was first report to quantify the degree of hemoperitoneum through ultrasonography and abdomen–pelvic CT and to measure the cut-off value of surgical treatment. To our knowledge, this was the only study to find out the association between the severity of extravasation and the possibility of surgery and the presence of liver-dome fluid as a risk factor of surgery.

### Limitations

This study had some limitations. First, the sample size is small, so large-scale research is needed. Second, this is a retrospective chart review which may have a selection bias. Third, the data are not from a single surgeon, although the indication for operative treatment was similar for all patients.

## Conclusion

PCDS fluid collection depth, the presence of liver-dome fluid, and the severity of contrast extravasation through ultrasonography and CT scan are good indicators for determining the management of hemoperitoneum resulting from CLCR in healthy women.

## Data Availability

The datasets used and/or analyzed during the current study are available from the corresponding author on reasonable request.
